# CONNECTING PROVIDER TO HOME: BRIDGING GAPS IN CARE

**DOI:** 10.1093/geroni/igz038.1823

**Published:** 2019-11-08

**Authors:** Melanie Weir, Gerardo Moreno, Rosaneli Loza, Lisa Desai, Chi Hong Tseng

**Affiliations:** 1 SCAN Health Plan, Long Beach, California, United States; 2 University of California, Los Angeles, Los Angeles, California, United States

## Abstract

When the physician has limited knowledge of the patient’s condition and functioning at home it may result in non-adherence to treatment plans, goals of care not being met and avoidable utilization. Connecting Provider to Home (CP2H) deployed teams of a social worker and community health worker to act as the eyes and ears of the doctor in patients’ homes and close the information gap in primary care. Study objectives were to 1) reduce unnecessary utilization, 2) increase provider and patient satisfaction, and 3) Improve communication between patient/caregiver and the healthcare team. A total of 416 adult patients were enrolled with a mean age of 76 years, and 58% were female. CP2H participants demonstrated statistically significant reductions in acute hospitalizations and ER use when compared to 700 controls. Acute hospitalizations were reduced by 216 and ER visits by 531 in the intervention group. The average per patient per year reduction in acute hospitalizations was 0.67. The average per patient reduction in ER use was 0.58. CP2H patients reported high levels of satisfaction and rated the program favorably. Stakeholder interviews found that physicians and staff believed the program improved clinical outcomes, provided valuable insight about patients’ social barriers to self-care and added value. CP2H study results provide evidence that social workers and community health workers can be successfully and cost-effectively incorporated into the primary care team to address patient needs and priorities, observe the patient in the home environment and assist the physician in adapting treatment plans to optimize patient care.

